# Evaluating Exercise as Evidence-Based Practice for Individuals with Autism Spectrum Disorder

**DOI:** 10.3389/fpubh.2016.00290

**Published:** 2017-02-07

**Authors:** Suzanna R. Dillon, David Adams, Leah Goudy, Melissa Bittner, Scott McNamara

**Affiliations:** ^1^Texas Woman’s University, Kinesiology, Denton, TX, USA

**Keywords:** evidence-based practice, exercise, autism spectrum disorder, systematic review, adapted physical education

## Abstract

**Background:**

The purpose of this study was to conduct a systematic review of the literature to evaluate empirical support for the use of exercise as an evidence-based practice (EBP) for individuals with autism spectrum disorder (ASD), aged 1–21 years, using the Adapted Physical Activity Taxonomy (APAT) (1).

**Method:**

A systematic review of research, published within the past 10 years and accessible in SPORTDiscus, ProQuest Nursing, Science Direct, ERIC, Ovid MEDLINE, and PsychINFO databases, was conducted following seven inclusion criteria. An initial 169 articles were identified of which 23 articles were found that met the inclusion criteria including implementation of an exercise intervention for participants diagnosed with ASD and utilization of an experimental/quasi experimental, correlational, single–subject, or qualitative research design. These 23 articles were evaluated using the APAT to determine the quality of the research and the strength of the recommendation in establishing exercise as an EBP.

**Results:**

Of the 23 articles evaluated, 17 employed an experimental/quasi experimental design, 1 article employed a correlational design, and 5 articles employed a single-subject design. Only one article (2) was found to meet the minimum overall quality indicator of moderate (i.e., Level 2) when evaluated on the APAT. In total, 13 of the 23 articles (57%) had method sections evaluated as weak, and 17 of the 23 articles (74%) had results sections evaluated as weak.

**Conclusion:**

From the findings of this systematic review, and in accordance with the *Every Student Succeeds Ac*t of 2015 (3) definition of an EBP, it appears that exercise can be considered an EBP for school-aged children with ASD. However, this recommendation is based solely on moderate evidence from one well-designed and well-implemented experimental study; therefore, generalization is still pending further similar findings. Recommendations for future research are offered.

Autism spectrum disorder (ASD) is defined by the American Psychiatric Association (APA) as a group of developmental disabilities causing significant delays in communication (e.g., limited expressive language) and social skills (e.g., difficulty with social reciprocity) and is associated with repetitive behavior (e.g., engages in hand-flapping) and stereotypical movements (e.g., body rocking) (4). Recent investigations of the motor and exercise patterns of individuals with ASD have also established motor development delays as an attribute of ASD (5–7). Individuals diagnosed with ASD will be categorized as one of three levels based on support needed: Level 1, requiring support; Level 2, requiring substantial support; and Level 3, requiring extreme support at all times (APA). These individual attributes and the levels of support needed by individuals with ASD may interfere with the development of age-appropriate motor skills and participation in exercise.

The physical benefits of exercise reported for children with ASD include improvements in cardiorespiratory functioning (8–10), motor skill performance (11), and muscular strength (10, 12), as well as a reduction in body mass index (13). Along with physical benefits, behavioral and cognitive functioning improvements have been reported. Exercise, as an intervention, has also been shown to reduce maladaptive behaviors (14, 15) and stereotypic behaviors (16) as well as increase on-task behaviors (17) and academic responding (e.g., participating in instructional tasks, asking, and answering questions) (18). Exercise has also been shown to improve academic achievement (19) and social skills (20). However, the research studies cited above provide varying definitions of exercise, utilize different methods to determine levels of heart rate or energy expenditure, and examine differing health-related fitness components. These research studies also involve participants across a wide age range and participants who include, but may not be exclusive to, individuals with ASD. These factors contribute to the difficulties of establishing exercise as an evidence-based practice (EBP). Furthermore, to date, no systematic review has been completed that examines the quality of the research or the strength of the recommendation needed to establish exercise as an EBP.

## Evidence-Based Practice

While the exact definition may vary between professions, EBP can generally be defined as an instructional strategy, intervention, or teaching program that is grounded in scientifically based research (21). Within legislation, the Individuals with Disability Education Improvement Act of 2004 lacks a definition but does imply that teachers use EBPs, mandating instructional interventions grounded in “scientifically based research,” when teaching students with disabilities (22). Conversely, the newly passed Every Student Succeeds Act of 2015 (ESSA) (3), after which the reauthorization of IDEA may be modeled, does clearly define evidence-based as:

an activity, strategy, or intervention that—(i) demonstrates a statistically significant effect on improving student outcomes or other relevant outcomes based on—(I) strong evidence from at least 1 well-designed and well-implemented experimental study; (II) moderate evidence from at least 1 well-designed and well-implemented quasi-experimental study; or (III) promising evidence from at least 1 well-designed and well-implemented correlational study with statistical controls for selection bias; or (ii) (I) demonstrates a rationale based on high-quality research findings or positive evaluation that such activity, strategy, or intervention is likely to improve student outcomes or other relevant outcomes; and (II) includes ongoing efforts to examine the effects of such activity, strategy, or intervention (22).

Prior to the Federal definition to guide educational practice, a number of organizations including the National Professional Development Center on Autism Spectrum Disorder (NPDC) and the National Autism Center (NAC) reported on EBPs used in school settings with children with ASD. Both organizations included exercise in their EBP reports but with differing results regarding the effectiveness of exercise, based on the taxonomies used for evaluation.

The NPDC defined EBP as interventions that have been proven through research to be effective and used their own criteria for evaluation when reviewing peer-reviewed research in scientific journals to reported on 27 EBPs for children with ASD (23). NPDC recognized exercise as an EBP and purported that exercise improves physical fitness, increases desired behaviors, and decreases inappropriate behaviors for children with ASD, aged 3–5 years (14, 18); and adolescents with ASD, aged 12 through 14 years (12, 24).

At the same time that the NPDC was releasing their report, the NAC released the *National Standards Project: Phase 2 Report* (25). For their report, the NAC adopted the definition of an EBP offered by Dr. David Sackett and his colleagues in *Evidence-Based Medicine: How to Practice and Teach EBM* (26) and systematically evaluated peer-reviewed research using a Scientific Merit Rating Scale and a Strength of Evidence Taxonomy. Within their *National Standards Project: Phase 2 Report* (25), the NAC identified 14 established EBPs, for children and young adults under the age of 22 years, but did not identify exercise within the established EBPs. Rather, the NAC identified exercise as an intervention with an emerging level of support and indicated that more high-quality studies were needed that consistently documents the effectiveness of exercise. The discrepancy between the conclusions drawn by the NPDC and the NAC, two leading organizations for research on children with ASD, makes the selection and implementation of EBPs problematic for teachers and researchers.

In order to gain scientific corroboration, there is a need to determine evidence for the establishment of exercise as an EBP for individuals with ASD. However, to date, no systematic review of the literature has been completed on exercise as an EBP using a taxonomy specific to the field of adapted physical activity or the discipline of kinesiology that educators could use to justify their use of exercise as an EBP. Therefore, the purpose of this study was to conduct a systematic review of the literature to evaluate empirical support for the use of exercise as an EBP for individuals with ASD, aged 1–21 years, using the Adapted Physical Activity Taxonomy (APAT) (1).

## Method

### Systematic Review Procedures

**Figure 1:**
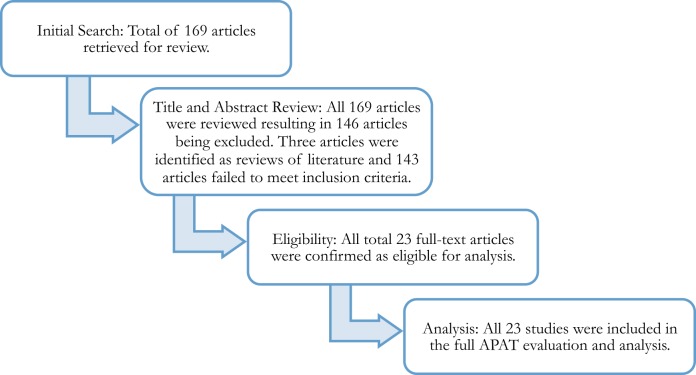
**Summary of systematic review process procedures**.

This systematic review of the literature (27) focused on the use of exercise as an intervention for children and youth with ASD. Prior to conducting the literature search, the reviewers unanimously agreed to (a) the operational definition for exercise for the study as “a subcategory of physical activity that is planned, structured, repetitive, and purposive to improve or maintain one’s physical fitness” (p. 250) (28) and (b) the minimum APAT (1) Quality of Study rating (i.e., Level 1 or Level 2) and Level of Recommendation (i.e., A or B) needed for the establishment of exercise as an EBP. These parameters, along with the inclusion criteria, guided the systematic review (see Figure 1 for an overview of the procedures).

#### Initial Search Procedure

Potential articles, published in the past 10 years, were initially located *via* online indexing system searches. The reference lists of the articles found through the online search were also manually searched for potential articles. The reviewers conducted an initial search of the literature using the indexing systems/research platforms of SPORTDiscus, ProQuest Nursing, Science Direct, ERIC, Ovid MEDLINE, and PsychINFO. Searches were conducted with search limiters of English language journals published within the last 10 years, and the keywords of exercise and ASD including the terms autism, Asperger’s syndrome, and PDD-NOS.

#### Criteria for Inclusion

The following seven inclusion criteria were selected by the five authors and required that articles be (a) published between January 2006 and April 2016; (b) published in English language journals; (c) located in periodical publications (i.e., books, unpublished papers, conference proceedings and book chapters were excluded); (d) involved implementation of an exercise intervention consistent with the adopted operational definition for exercise; (e) provided a clear description of the participants as individuals diagnosed with ASD, to include participants with autism, Asperger’s syndrome and Pervasive-Developmental Disorder—Not Otherwise Specified (i.e., studies with participants with ASD who were diagnosed with other/secondary disabilities were excluded); (f) included participants between the ages of 1 and 21 years; and (g) utilized an experimental/quasi experimental, correlational, single-subject, or qualitative research design (i.e., systematic reviews and meta-analyses were excluded). Only articles that met these criteria were eligible for evaluation.

#### Title and Abstract Review

Research studies, identified through the initial search procedure, were then evaluated using a three-step process. First, reviewers conducted a title and abstract review on the potential studies identified in the initial search to confirm studies met inclusion criteria. Articles identified as reviews of literature were excluded but the reference lists from these articles were examined for additional potential articles. In the second step, articles meeting the inclusion criteria were then independently evaluated by one of the authors using the APAT. Finally, the second author independently evaluated each article to confirm agreement on the evaluation of the article as a Level I, II, or III.

### Instrumentation

The APAT Quality of the Study and Letter of Recommendation (1) were used as the decision-making tool to rate the quality of each individual study and the strength of the recommendations for each of the identified articles. Designed to address four types of research designs (i.e., experimental/quasi experimental, single-subject, correlational, qualitative), the APAT contains five evaluation domains (i.e., introduction, method, results, discussion, and others) with quality indicators delineated within each domain. See Tables 1–3 for additional details regarding the content evaluated within each domain. These domains guide the evaluation of the article and provide an APAT Quality of the Study rating (i.e., Level 1 = strong; Level 2 = moderate; Level 3 = weak). The APAT also follows a standardized decision-making process to determine an APAT Level of Recommendation (i.e., A, B, and C Levels of Recommendation) for the research reported within the article. Articles evaluated as having a Level of Recommendation of A are those studies with outcomes that meet at least one of the following: (a) “result in significant value that can be applied to educational, recreational, and disability sport settings”; (b) have “consistent findings from at least two good-quality randomized controlled trials or a systematic review/meta-analysis”; or (c) have a “validated intervention decision relevant to a disability population” [APAT: Level of Recommendation (Part II)].

**Table 1:** **Exercise intervention studies for individuals with autism spectrum disorder (ASD): study design, participant information, intervention type, and outcomes**.

Study	Study design	Participant information		Intervention type	Outcomes
		Sample size	Age range (years)		
Anderson-Hanley et al. (29)	Quasi-experimental	*N* = 12	CA = 10–18	Exergaming cybercycling	RSB, EF: decreases in repetitive behaviors and improvements in executive functioning following exergaming
		*N* = 22	CA = 8–21		
Arzoglou et al. (30)	Experimental	*N* = 10	CA = 16	Greek dance training program	SRF: improvements in measures of neuromuscular coordination following participation in Greek dance intervention program
Bahrami et al. (31)	Experimental	*N* = 30	CA = 5–16	Kata (karate)	RSB: decreases in stereotypical behavior after intervention
Chan et al. (32)	Experimental	*N* = 46	CA = 6–17	Nei Yang Gong (Chinese mind-body exercise) versus progressive muscle relaxation	SBI, RSB: greater improvements in self-control and reductions in typical autistic symptoms and daily emotional and behavioral problems of children with ASD after Nei Yang Gong intervention than progressive muscle relaxation
Fragala-Pinkham et al. (9)	Single subject	*N* = 16	CA = 6–11	Group aquatic exercise program	HRF: improvements in cardiorespiratory endurance after a group aquatic intervention with a high adult to child ratio and specific goals to maintain training heart rates
Fragala-Pinkham et al. (24)	Quasi-experimental	*N* = 12	CA = 6–12	Aquatic exercise program	HRF, MSD: no significant between-group changes found for swimming skills, cardiorespiratory endurance, muscular endurance, and mobility skills. Within-group improvements for swimming skills were found for the intervention group
Goodarzi and Hemayattalab (33)	Experimental	*N* = 50	CA = 8–10	6-month program of weight bearing exercises (three sessions per week) and/or the addition of dietary calcium rich food (250 mg calcium/serving)	BD: greater increases in bone mineral density with additional weight bearing exercise and calcium supplementation than control
Hawkins et al. (34)	Single subject	*N* = 2	CA = 7–11	5-week equine-assisted therapy program	SRF, HRF, MSD: moderate to large gains in body coordination, strength and agility, and overall gross motor skills as a result of participation in an equine-assisted therapy intervention
Hillier et al. (35)	Quasi-experimental	*N* = 18	CA = 13–27	8-week physical exercise and relaxation program	SBI: significant reductions in salivary cortisol levels and self-reported anxiety measure following intervention
Koenig et al. (36)	Experimental	*N* = 48	CA = 5–12	16-week get ready to learn classroom yoga program	SBI, RSB: significant improvements in classroom behaviors as measured by the ABC-community scored by teachers but no significant difference as scored by parents following the intervention
Lee and Porretta (37)	Single subject	*N* = 3	CA = 3–6	16-session physical activity program focused on object manipulation and locomotor activities	RSB, TOT: locomotor activities found to be effective in decreasing stereotypic behaviors and increasing time-on-task when compared to object manipulation activities
Lourenco et al. (38)	Experimental	*N* = 16	CA = 4–10	20-week trampoline training program	MSD, HRF: significant improvements in motor performance for intervention group. No significant differences for body mass index
Morrison et al. (39)	Single subject	*N* = 4	CA = 10–21	Antecedent physical exercise program (e.g., stationary bike, therapy ball) prior to instruction	SBI: antecedent exercise and access to leisure items reduced problem behaviors decreased during and post-intervention
Movahedi et al. (40)	Experimental	*N* = 30	CA = 5–16	14-week Kata technique training program	SBI: significant improvements in social interactions for the intervention group
Neely et al. (41)	Single subject	*N* = 2	CA = 7–8	Antecedent physical exercise prior to instruction	RSB, TOT: increases in academic engagement and reduced levels of stereotypy during the instructional sessions, which followed antecedent physical exercise
Pan (2)	Quasi-experimental	*N* = 16	CA = 6–9	20-week water exercise swimming program	MSD, SBI: improved aquatic skills and decreased the total antisocial behaviors after intervention
Pan (12)	Correlation	*N* = 95	CA = 14	16-week physical education program	SBI, HRF: steps per minute for students with ASD were significantly lower than their peers without disabilities. Intervention features including physical activity content, lesson location, and instructor-related characteristics were associated with student MVPA. Social interactions were positively related to physical activity levels of students with ASD
		With ASD (*n* = 19) and without ASD (*n* = 76)			
Pan et al. (42)	Experimental	*N* = 22	CA = 6–12	12-week physical activity intervention focused on table tennis and body movement skills	HRF, SRF, EF: significant interaction effects and intervention induced improvements for the intervention group on measures of manual coordination, body coordination, strength, and agility as well as executive functioning
		Control (*n* = 11), Intervention (*n* = 11)			
Pitetti et al. (13)	Quasi-experimental	*N* = 10	CA = 14–19	9-month treadmill-walking program	HRF: significant increases in mean monthly treadmill-walking program frequency, speed, elevation, and calories expended along with a reduction in BMI as a result of the intervention
Ringenbach et al. (43)	Experimental	*N* = 10	CA = 8–16	Assisted cycling therapy (ACT), voluntary cycling (VC), and no cycling (NC)	EF: significant improvements in inhibition with improvements in cognitive planning and set-switching approached significance after a single session of ACT. No improvements were found in inhibition, cognitive planning, or set-switching following the VC or NC sessions. Exercise perception improved after the VC session but did not change after the ACT or NC sessions
Rosenblatt et al. (44)	Quasi-experimental	*N* = 24	CA = 3–16	8-week multimodal yoga, dance, and music therapy program based on the relaxation response	SBI: significant differences on the BASC-2 behavioral symptom index, with positive non-significant impacts on the BASC-2 externalizing scale and internalizing scale and ABC-irritability scale following intervention
Todd and Reid (45)	Single subject	*N* = 3	CA = 15–20	Snowshoe/walk/jog program, twice a week for 30 min for 28 sessions	HRF: increases in distance snowshed/walked/jogged and decreases in need for verbal cueing to persist in physical activity sessions
Wuang et al. (46)	Experimental	*N* = 60	CA = 6–8	20-week simulated developmental horse-riding program	MSD: improved motor performance and sensory integrative functions post-intervention that were sustained for at least 6 months

*SBI, social and behavioral issues; RSB, repetitive and stereotypical behaviors; HRF, health-related fitness; MSD, motor skill development; SRF, skill-related fitness; EF, executive functioning; TOT, time-on-task; BD, bone density*.

**Table 2:** **Adapted Physical Activity Taxonomy quality indicator ratings for correlational research reviewed**.

Quality indicators	Reference
	Pan (12)
**Introduction**	
Hypothesis/research question stated, theory or conceptual model, significance and need, alignment of purpose, solutions and challenges, and literature support	1
**Method**	
Research design appropriately aligns with the hypothesis/research question, instrument currently validated reliable within the target population, appropriate measures are used to control for participant and researcher bias, data collection conducted throughout the treatment; if appropriate substantial baseline obtained, participants reflect the intended study, population is adequately represented description of inclusion/exclusion criteria, sampling technique(s), replication, description of settings, IV and DV explained, confidentiality, fidelity	3
**Results**	
Percent agreement between observers is >90%, or coefficient *r* is >0.7, analyses of raw data are clearly described, effect size is provided, confidence intervals are presented for reliability coefficients and sample statistics, univariate measures are used only when appropriate, reliability and validity interpretations are very detailed	3
**Discussion**	
Discussion of results clearly address the hypothesis/research question, findings compared to prior research, limitations defined, recommendations, representativeness addresses target population, and other possible issues	2
**Other**	
Complete listing of references pertinent to the study concept, appendices provided when appropriate	1
**Level of quality**	3
**Letter of recommendation**	A

**Table 3:** **Adapted Physical Activity Taxonomy (APAT) quality indicator ratings for experimental/quasi experimental research reviewed**.

Quality indicators	Reference																
	Anderson-Haley et al. (29)	Arzoglou et al. (30)	Bahrami et al. (31)	Chan et al. (32)	Fragala-Pinkham et al. (9)	Fragala-Pinkham et al. (24)	Goodarzi and Hemayattalab (34)	Hillier et al. (35)	Koenig et al. (36)	Lourenco et al. (38)	Movahedi et al. (40)	Pan (2)	Pan et al. (42)	Pitetti et al. (13)	Ringenbach et al. (43)	Rosenblat et al. (44)	Wuang et al. (46)
Introduction	1	1	2	3	2	2	3	1	1	1	1	1	1	3	3	1	1
Method	2	3	2	2	3	3	3	3	1	3	1	2	2	3	3	2	2
Results	3	3	3	3	3	3	3	3	3	3	3	2	3	3	3	3	3
Discussion	1	3	3	2	1	1	3	1	1	3	3	1	1	1	3	1	1
Others	1	1	1	1	1	1	1	1	1	1	1	1	1	1	1	1	1
Level of quality	3	3	3	3	3	3	3	3	3	3	3	2	3	3	3	3	3
Letter of recommendation	A	A	A	A	A	A	A	A	A	A	A	A	A	A	A	A	A

*Details specific to each of the APAT quality indicators for experimental and quasi-experimental research have been omitted for table brevity*.

### Inter-Rater Agreement

The authors of this article independently assessed all the titles and abstracts to determine whether the studies met the criteria for inclusion using a dichotomous scale (yes or no). In instances of disagreement, articles were re-assessed until an inter-rater agreement of 100% was reached. All of the articles were also independently evaluated by at least two of the current authors using the APAT. There were no instances of disagreement during the evaluation using the APAT; hence, there was 100% inter-rater consensus.

## Results

The initial search of the literature identified 169 exercise-based intervention studies targeting individuals with ASD, aged 1–21 years (see Figure 1). Of the 169 articles identified through the initial search, 146 articles were excluded from further analysis as a result of failing to meet 1 of the 7 inclusion criteria. Three of the 146 articles were excluded because they were identified as reviews of literature in the title and abstract review. The references for these articles were examined for additional articles. None of the potential articles found on the reference list met the publication year inclusion criteria. See Figure 1 for a summary of the systematic review process.

The resulting 23 articles remained for analysis and were evaluated using the APAT, with 17 articles evaluated as experimental/quasi experimental, 1 article as correlational, and 5 articles as single-subject designs. Table 1 presents a summary of each article reviewed including the study design, participant information, intervention type, and study outcomes. The studies evaluated employed fitness-focused exercise (*n* = 8), aquatics (*n* = 3), karate and martial arts training (*n* = 3), motor skills programming (*n* = 3), yoga (*n* = 2), dance (*n* = 2), equine-assisted programming (*n* = 2), relaxation training (*n* = 2), and exergaming (*n* = 1) interventions. These exercise interventions implemented attempted to address ASD-related issues including social and behavioral issues (*n* = 8), repetitive and stereotypical behaviors (*n* = 5), health-related fitness (*n* = 4), skill development (*n* = 4), skill-related fitness (*n* = 3), cognitive functioning (*n* = 2), and time-on-task (*n* = 2). While the interventions were implemented with participants with ASD from 4 to 27 years of age, a majority of the studies (74%) targeted adolescents with ASD as participants.

In addition to the descriptive summary table, a summary of the APAT evaluation ratings for the 23 articles reviewed are presented, by research design, in Tables 2–4. A closer examination of the articles presented in Tables 2–4 reveals that 13 of the 23 articles (57%) had method sections evaluated as weak (i.e., Level 3 rating), and 17 of the 23 articles (74%) had results sections evaluated as weak. This weak rating is problematic as the method and results sections provide details essential to quality design and study replication as well as the research findings. Additionally, 10 of the 23 articles (43%) were published without a clearly stated hypothesis or theory.

**Table 4:** **Adapted Physical Activity Taxonomy quality indicator ratings for single-subject design research reviewed**.

Quality indicators	Reference				
	Hawkins et al. (34)	Lee and Porretta (37)	Morrison et al. (39)	Neely et al. (41)	Todd and Reid (45)
**Introduction**					
Hypothesis/research question stated, theory or conceptual model, significance and need, alignment of purpose, solutions and challenges, and literature support	1	3	3	3	3
**Method**					
Research design aligns with the hypothesis/research question, data collection substantiates trustworthiness, baseline if needed, adequate representation of population, inclusion criteria, information for replication, description of setting, sample techniques, intervention and conditions explained, participant information defined and clear, threats to internal validity addressed	3	3	3	3	2
**Results**					
Percent agreement between observers is ≥80%, or coefficient r is ≥0.7, raw data clearly described, pattern of experimental control defined, 3 or more different experimental effects over 3 different periods presented	2	3	1	1	1
**Discussion**					
Discussion of results clearly address the hypothesis/research question, findings compared to prior research, limitations defined, recommendations, inclusion and exclusion criteria, generalizability, DV supported, IV practical and cost effective	1	3	3	3	3
**Other**					
Complete listing of references pertinent to the study concept, appendices provided when appropriate	1	3	3	3	3
**Level of quality**	3	3	3	3	3
**Letter of recommendation**	A	A	A	A	A

As can be observed from the aforementioned tables, only 1 article (2) from the 23 articles reviewed was found to meet the minimum overall quality indicator of Level 2 when evaluated on the APAT. In his research, Pan (2) examined the effects of a water exercise swimming program on the aquatic skills and social behaviors of children, aged 6–9 years, diagnosed with ASD. The participants were split into two groups, Group A (*n* = 8) and Group B (*n* = 8). Group A received water exercise swimming program in the first 10-week phase followed by a week break and then a 10-week phase of baseline treatment/activity. Group B received treatments in reverse order. Pan (2) found improved aquatic skills as well as a decrease in the frequency of antisocial behaviors (e.g., spinning, rocking, and delayed echolalia) in children with ASD. While Pan (2) was able to report significant improvements in aquatic skills, sustainability of improvements, and significant decreases all antisocial behaviors, the research article as a whole was evaluated as having only moderate strength of quality. More specifically, when evaluated *via* the APAT, the Pan article (2) was strong (i.e., Level 1) in the introduction, discussion, and other section but only moderate in the method section (e.g., sampling technique described but not replicable), and the results section (e.g., reliability and validity interpretation lacked detail) producing a moderate overall rating.

## Discussion

The purpose of the current study was to conduct a systematic review of the literature to evaluate empirical support for the use of exercise as an EBP for individuals with ASD, aged 1–21 years. A total of 23 articles were evaluated. Based on the findings of this systematic review, and utilizing the newly enacted ESSA (2016) definition of an EBP, it appears that exercise can be considered an EBP for school-aged children with ASD. However, this recommendation is based solely on moderate evidence from one well-designed and well-implemented experimental study (2), therefore, generalization is still pending further similar findings. These current systematic review findings are consistent with that of Lang et al. (47) who reported a limited literature base and called for additional high-quality research, especially studies using a strong experimental design, which could assist educators in developing effective programing for individuals with ASD.

As researchers move forward with designing and conducting the research to further establish the evidence base, they need to be mindful of the research-to-practice gap. It has been established that interventions that are too narrowly focused, complex, difficult to implement or costly; or interventions that do not meet the perceived needs of the community (48) perpetuate the gap and impede the process of converting empirically supported discoveries into routine educational practices. For example, within his study, Pan implemented a 10-week water exercise swimming program with multiple swimming instructors (i.e., a one instructor-to-two student instructional setting) and two 90-min instructional sessions per week. This design, while ideal for research purposes, is not easily implemented in PK-12 settings where instructional sessions are often shorter, staff ratios higher, and pools present in only some of the schools. Furthermore, researchers must take care to report all of the information critical to their research design and findings, such as those quality indicators outlined in the APAT (1), in order to improve the quality of the study and strengthen the resulting recommendations. We recognize that this can be a daunting task, but it is essential if exercise, or any other instructional strategy, intervention, or teaching program is to be firmly established as an EBP (21) for use with children and youth with ASD.

## Author Contributions

The first author, SD, was responsible for designing and guiding the systematic review and was responsible for the preparation/writing of the manuscript. The remaining authors (DA, LG, SM, and MB) were involved in the systematic review of the literature including the evaluation of the 23 articles using the Adapted Physical Activity Taxonomy. The second (DA) and third (LG) authors provided significant assistance to the first author in the development of the manuscript. The fourth (SM) and fifth (MB) authors also provided editorial assistance in the development of the manuscript but to a lesser extent than the second and third authors.

## Conflict of Interest Statement

The authors declare that the research was conducted in the absence of any commercial or financial relationships that could be construed as a potential conflict of interest.
